# Exosomes Derived from Mg-Preconditioned Bone Mesenchymal Stem Cells Promote Angiogenesis and Osteogenesis for Osteonecrosis Treatment

**DOI:** 10.3390/ma18204687

**Published:** 2025-10-13

**Authors:** Long Li, Luyao Cheng, Yuhan Du, Yuyang Zhang, Zetao Wang, Yangyi Nie, Jing Long, Cairong Li, Yuanchi Zhang, Yuxiao Lai, Wei Zhang

**Affiliations:** 1 Centre for Translational Medicine Research and Development, Shenzhen Institutes of Advanced Technology, Chinese Academy of Sciences, Shenzhen 518055, China; 2 University of Chinese Academy of Sciences, Beijing 101408, China; 3 National Innovation Center for Advanced Medical Devices, Shenzhen 518131, China; 4 State Key Laboratory of Biomedical Imaging Science and System, Shenzhen 518055, China

**Keywords:** exosome, BMSCs, magnesium, angiogenesis, osteogenesis

## Abstract

Steroid-induced osteonecrosis of the femoral head (SONFH) is a common and refractory orthopedic disorder, often resulting from prolonged or high-dose glucocorticoid use that impairs bone repair and vascularization. The critical impact of exosomes derived from bone mesenchymal stem cells (BMSCs) in bone regeneration has drawn increasing attention. In this study, we developed a novel type of exosomes derived from Magnesium-preconditioned BMSCs (Mg-Exos) and evaluated their therapeutic potential. In vitro experiments demonstrated that Mg-Exos effectively counteracted Dex-induced impairment in the angiogenic function of human umbilical vein endothelial cells (HUVECs) and the osteogenic differentiation of BMSCs. These findings highlight the promise of Mg-Exos as a potential cell-free therapeutic strategy for SONFH, acting through the concurrent enhancement of vascularization and bone formation. Consequently, this work lays a solid foundation for the future application of Mg-Exos in treating SONFH.

## 1. Introduction

Glucocorticoids (GCs) are widely employed in clinical practice as a conventional therapeutic agent for various conditions, including autoimmune diseases and acute infectious diseases. However, both long-term and short-term high-dose administration of GCs is associated with multiple adverse effects [1]. GC-induced osteonecrosis, also known as steroid-induced osteonecrosis of the femoral head (SONFH), is a common GC-triggered disabling condition and represents the most prevalent form of non-traumatic osteonecrosis [2]. SONFH is a complex pathological process involving multiple mechanisms under GC influence. Crucially, impaired blood supply due to endothelial damage and reduced osteogenic capacity constitute major contributing factors to femoral head collapse in SONFH [3,4,5,6]. Current clinical management of SONFH primarily relies on surgical interventions such as core decompression, bone grafting, and osteotomy [7]. While these approaches yield some therapeutic benefits, they fail to fundamentally reverse femoral head ischemia or prevent subsequent collapse. Consequently, developing strategies capable of simultaneously repairing vascular endothelial cells and damaged bone tissue during the early stages of SONFH represents a potentially effective therapeutic approach.

With advances in technology, stem cell therapy has garnered significant attention. Stem cells possess self-renewal potential and multipotent differentiation capacity, holding substantial promise for treating diverse diseases [8,9]. Bone marrow-derived mesenchymal stem cells (BMSCs), the most commonly utilized stem cells in bone tissue engineering, play a vital role in maintaining bone homeostasis. They can differentiate into multiple lineages, including osteoblasts and endothelial cells [10], and secrete numerous bioactive factors via paracrine pathways to mediate intercellular communication, thereby promoting bone repair and regeneration [11,12,13,14]. Despite these advantages, stem cell-based therapies face several formidable challenges that hinder their clinical translation. Key concerns include the risk of immune rejection, potential tumorigenicity, which requires careful evaluation or unintended differentiation, and low survival and engraftment rates of transplanted cells at the injury site. Given these limitations, there is a growing impetus to identify alternative cell-free therapeutic strategies [15]. In recent years, extracellular vesicles (EVs), particularly exosomes, have gained significant attention as a promising novel approach [16,17].

Exosomes are cell-secreted extracellular vesicles with a diameter of 30–150 nm. They facilitate intercellular communication and signal transduction by delivering cargoes such as proteins, mRNAs, and miRNAs [18,19,20]. Exosomes are formed through the budding of the endosomal membrane and subsequently released into the extracellular space, and recognized and internalized by recipient cells, thereby exerting biological functions similar to their parent cells. Crucially, they lack the immunogenicity associated with stem cells, effectively mitigating concerns such as immune rejection and tumorigenicity [21]. Studies demonstrate that exosomes derived from BMSCs can deliver cargoes like miRNAs and proteins. By modulating various signaling pathways, BMSC-Exos influence endothelial cells and osteoblasts, promoting angiogenesis and bone repair [22,23,24,25].

The yield and cargo composition of exosomes secreted by cells are influenced by both the cellular tissue origin and the physicochemical stimuli applied to the cells. Preconditioning stem cells with various physicochemical strategies, such as hypoxia, cytokine/ion treatment and so on, offers a promising strategy to enhance the biological functions and therapeutic potential of their derived exosomes [26,27,28,29,30,31]. Magnesium ions (Mg^2+^), essential cations in the human body, exert diverse biological functions. By regulating the osteogenic immune microenvironment via M2 macrophage polarization, and upregulating the expression of key osteogenic genes such as bone morphogenetic protein-2 (BMP2), Runt-related transcription factor 2 (Runx2), and osteoprotegerin (OPG), Mg^2+^ can promote osteogenic differentiation. Mg^2+^ can also stimulate angiogenesis by regulating endothelial cell adhesion, proliferation, and migration, and enhancing vascular endothelial growth factor (VEGF) expression [32,33,34,35]. Furthermore, studies report that Mg^2+^-stimulated macrophages promote BMSC osteogenic differentiation by perturbing miR-381 within secreted exosomes through autophagy-dependent polarization. This suggests that Mg^2+^ can enhance osteogenic function by stimulating cells and altering the miRNA cargo of exosomes [36]. Therefore, exosomes derived from Mg^2+^-stimulated BMSCs (Mg-Exos) hold significant therapeutic promise for SONFH by promoting both osteogenesis and angiogenesis.

## 2. Materials and Methods

### 2.1. Cell Culture

rBMSCs and hBMSCs (ATCC, PCS-500-012) were cultured in α-MEM (Gibco, CA, USA) containing 10% fetal bovine serum (FBS, Gibco, CA, USA) and 1% penicillin/streptomycin (P/S, Gibco, CA, USA). Osteogenic differentiation was induced by replacing the standard culture medium with osteogenic induction medium, which consisted of α-MEM complete medium supplemented with 10 mmol L^−1^ β-glycerophosphate, 50 µg mL^−1^ vitamin C, and 10 nmol L^−1^ dexamethasone (Dex; Sigma-Aldrich, St. Louis, MO, USA). The complete culture medium was replaced with adipogenic induction medium (Oricell, Guangzhou, China) for adipogenic induction. Human umbilical vein endothelial cells (HUVECs, ATCC, Manassas, VA, USA, PCS-100-013) [37] were cultured in complete DMEM medium. All cells were maintained at 37 °C in a humidified incubator with 5% CO_2_, with medium refreshed every two days, and cells were passaged or cryopreserved upon reaching 90% confluency.

### 2.2. Isolation and Characterization of rBMSCs

All animal procedures were approved by the Animal Care and Use Committee of the Shenzhen Institutes of Advanced Technology (SIAT), (Approval number: SIAT-IACUC-201016-YGS-LYX-A1469, 20 October 2020). rBMSCs were isolated from 6 to 8-week-old SD rats (Guangdong Medical Laboratory Animal Center, Guangzhou, China). After aseptically dissecting the femurs and tibias, bone marrow was flushed with serum-free α-MEM, followed by filtering through a 70-μm cell strainer (Falcon, Cary, NC, USA), and treated with red blood cell lysis buffer (Solarbio, Beijing, China). The cells were cultured in complete α-MEM medium. The medium was replaced after 24 h and subsequently every two days until confluency, followed by passaging. rBMSCs at passages 3 to 5 were used for subsequent experiments.

When the cells were cultured to the third generation, flow cytometry was used to identify the marker proteins of the cells. rBMSCs were digested, resuspended, and stained with antibodies against CD29 (eBioscience, San Diego, CA, USA)/CD90 (BioLegend, San Diego, CA, USA)/CD45 (eBioscience, San Diego, CA, USA) [38,39]. Unstained cells served as controls. After incubation in the dark at 4 °C for 30 min, cells were washed, resuspended, and analyzed by flow cytometry.

### 2.3. Isolation of Exosomes

When rBMSCs reached approximately 70% confluence, the medium was replaced with α-MEM containing 10% exosome-depleted serum (System Biosciences, Palo Alto, CA, USA), 1% P/S, and supplemented with either 10 mM MgCl_2_ or no Magnesium. Cells were then incubated at 37 °C for an additional 48 h. After incubation, exosomes were isolated from the culture supernatant by differential ultracentrifugation. The conditioned media were collected and centrifuged at 300× *g* for 10 min at 4 °C, and then the supernatant was carefully transferred to a new tube, followed by centrifuging at 2000× *g* for 10 min, 10,000× *g* for 30 min, and 110,000× *g* for 70 min at 4 °C. The resulting pellet was washed by resuspension in PBS and recentrifuged at 110,000× *g* for 70 min at 4 °C. The final pellet was then resuspended in a small volume of PBS. Subsequently, the resuspended exosomes were filtered through a 0.22-μm filter for sterilization and removal of large aggregates. Finally, the purified exosomes were aliquoted and stored at −80 °C.

### 2.4. Characterization and Internalization of Exosomes

Exosomes were characterized using TEM, Nano-Flow cytometry, and Western blot (WB). The exosomes were loaded onto copper grids, subjected to negative staining with uranyl acetate, and imaged under a transmission electron microscope (TEM, FEI Tecnai G2 12, Hillsboro, OR, USA) operating at 100 kV to observe their morphology. The particle size distribution and concentration of the exosomes were determined with the Flow NanoAnalyzer U30E (NanoFCM, Xiamen, China). Western blot analysis was conducted to detect the exosomal marker proteins CD63 (abcam, ab217345, Cambridge, UK), TSG101 (abcam, ab125011), and HSP70 (Beyotime, AF1156, Shanghai, China), respectively. After labeling the exosomes with PKH26 (Sigma, MIDI26, Shanghai, China), they were co-cultured with cells, and the internalization of the labeled exosomes was visualized and captured using a confocal laser scanning microscope (Zeiss, LSM900, Oberkochen, Germany), serving as qualitative documentation of cellular uptake.

### 2.5. Cell Proliferation

Cell proliferation was assessed using the CCK-8 kit. Cells were plated into a 96-well plate at 5000 cells per well. After cell adherence, the medium was replaced with conditioned medium containing dexamethasone and exosomes at various concentrations. At the designated time points of 1, 2, and 3 days, the conditioned medium was changed to the detection solution containing CCK-8 reagent (MIKX, Shenzhen, China). The plate was then incubated at 37 °C for 1 h, and the optical density (OD) at 450 nm was measured.

### 2.6. Wound Healing Assay

Cell migration was evaluated using a wound healing assay. HUVECs (3 × 10^4^ cells per well) were seeded into ibidi Culture-Insert 2 Wells positioned in 12-well plates and cultured until reaching 100% confluency. The inserts were subsequently removed. The complete medium was then exchanged for serum-free medium. Migration ability was quantified based on the scratch width, determined via microscopic measurement at 24 h time points. 3 random fields per replicate were analyzed by ImageJ (1.54g).

### 2.7. Transwell Migration Assay

The impact of different treatment conditions on endothelial cell migration was assessed using Transwell plates (Corning, CLS3422-48EA, Corning, NY, USA). Serum-starved HUVECs were seeded in the upper chamber at 40,000 cells per well. Dex, Exos, and Mg-Exos were added to the lower chamber according to experimental groups. Following 24 h of co-culture, cells on the membrane were fixed. The membrane was stained with 0.1% crystal violet (Beyotime, C0121, Shanghai, China), and unbound dye was removed by washing with PBS. Non-migrated cells on the upper surface were gently removed using a cotton swab. After thorough washing, the membrane was observed and imaged under a microscope. Manual counting was used for Transwell assays.

### 2.8. Tube Formation Assay

Matrigel (Corning, 356234, Corning, NY, USA) was mixed with DMEM medium at a ratio of 1:1. Then, 10 μL of the mixture was applied to ibidi culture slides and allowed to polymerize at 37 °C for 30 min. Subsequently, a 50 μL cell suspension (density: 1.2 × 10^4^ cells per well) was seeded onto the polymerized Matrigel. After 6 h of incubation, tube formation was visualized and imaged using a microscope. ImageJ with the AngioTool plugin was used for tube formation.

### 2.9. ALP Staining

BMSCs were plated in 12-well plates at 5 × 10^4^ cells per well, and cultured overnight. Following 14 days of osteogenic induction, the cells were fixed and subjected to alkaline phosphatase (ALP) staining using a BCIP/NBT kit (Beyotime, Shanghai, China) for 30 min, with the results then examined under a microscope.

### 2.10. ARS Staining

Mineralization of BMSCs was assessed by Alizarin Red S (ARS) staining. Cells were cultured and inducted using the same method as described in the ALP staining above. On day 21 post-induction, cells were fixed and stained with ARS. Following two washes with ddH_2_O, mineralized nodules were visualized microscopically. For quantification, ARS was solubilized in 10% cetylpyridinium chloride (CPC), and absorbance was measured at 562 nm.

### 2.11. RT-qPCR Analysis

Osteogenic and angiogenic gene expression was analyzed by RT-qPCR. At specified time points after treatment with Mg-Exos or Exos, total RNA was extracted using an Axygen RNA-250 kit (Silicon Valley, CA, USA). and reverse-transcribed to cDNA with PrimeScript™ RT Mix (Takara, San Jose, CA, USA). qPCR amplification was conducted in 10 μL reactions containing SYBR Premix Ex TaqII (Takara, San Jose, CA, USA) and specific primers (Table S1) on a Roche Light Cycler 96 system. and prevalidated primers (Table S1) on a Light Cycler 96 system (Roche, Basel, Switzerland). Expression levels were normalized to GAPDH and calculated via the 2^−ΔΔCt^ method.

### 2.12. Statistical Analysis

Statistical analyses were conducted using GraphPad Prism 9 (San Diego, CA, USA). Data represent mean ± SD from 3 independent replicates. Multi-group comparisons utilized one-way ANOVA with post hoc testing. Statistical significance was defined as * *p* < 0.05.

## 3. Results and Discussion

### 3.1. Isolation and Characterization of rBMSCs

**Figure 1:**
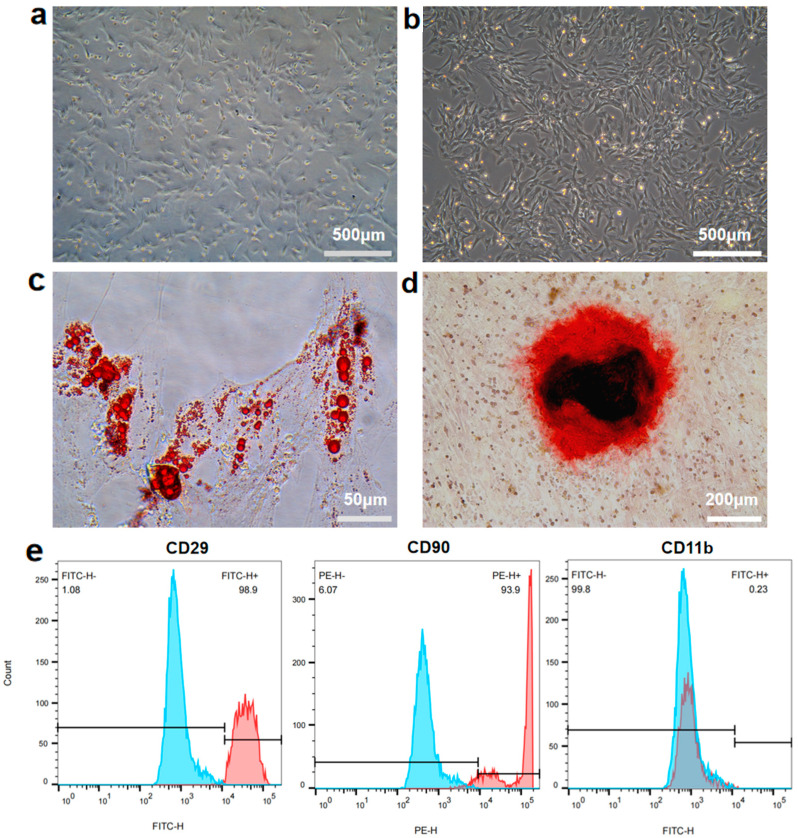
Characterization of rBMSCs. (**a**,**b**) Morphology of rBMSCs of the P1 generation (**a**) and P5 generation (**b**). (**c**) Oil Red O staining of adipogenically induced rBMSCs. (**d**) Alizarin Red S staining of osteogenically induced rBMSCs. (**e**) Flow cytometry of the cell surface marker on rBMSCs. The control group was presented as the blue curve and the test groups were presented as red curves.

Bone marrow mesenchymal stem cells (BMSCs) are an ideal choice for clinical bone repair due to their ability to participate in multiple processes, including angiogenesis and osteogenesis, through paracrine mechanisms [22,40]. In this study, rat bone marrow tissue was selected as the source of BMSCs. The isolated rBMSCs exhibited the typical spindle-shaped morphology. Upon reaching near confluence, the cells were arranged in an orderly and dense pattern, showing swirling and radial aggregation in their growth (Figure 1a,b). Following two weeks of adipogenic induction, Oil Red O staining showed the presence of red lipid droplets under the microscope (Figure 1c). Alizarin Red S staining revealed the formation of red mineralized nodules after osteogenic induction for 2 weeks (Figure 1d), demonstrating that the extracted rBMSCs possessed multidirectional differentiation potential into adipogenic and osteogenic lineages. Flow cytometry analysis indicated that the rBMSCs highly expressed the BMSC-specific surface markers CD29 (96.9%) and CD90 (93.9%), while CD45 expression was negative (Figure 1e). These results confirmed the successful extraction of rBMSCs from rat bone marrow tissue.

### 3.2. Characterization and Internalization of Exosomes

**Figure 2:**
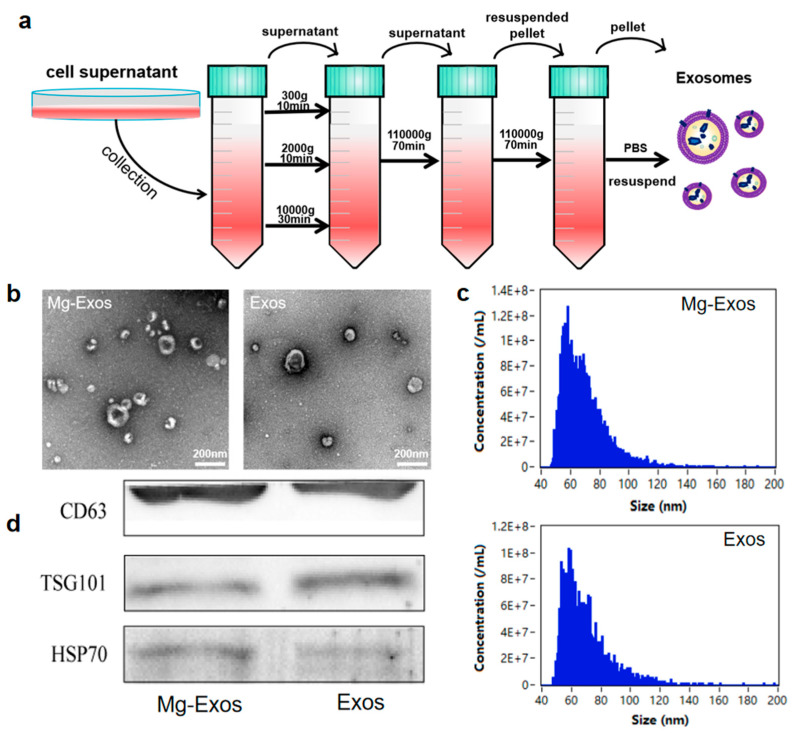
Isolation and characterization of Mg-Exos. (**a**) Schematic illustration of exosome isolation. (**b**) Representative TEM images of Exos. (**c**) Particle concentration and size distribution of Exos were determined via NanoFCM. (**d**) WB analysis of exosome surface markers CD63, TSG101, and HSP70. *n* = 3.

**Figure 3:**
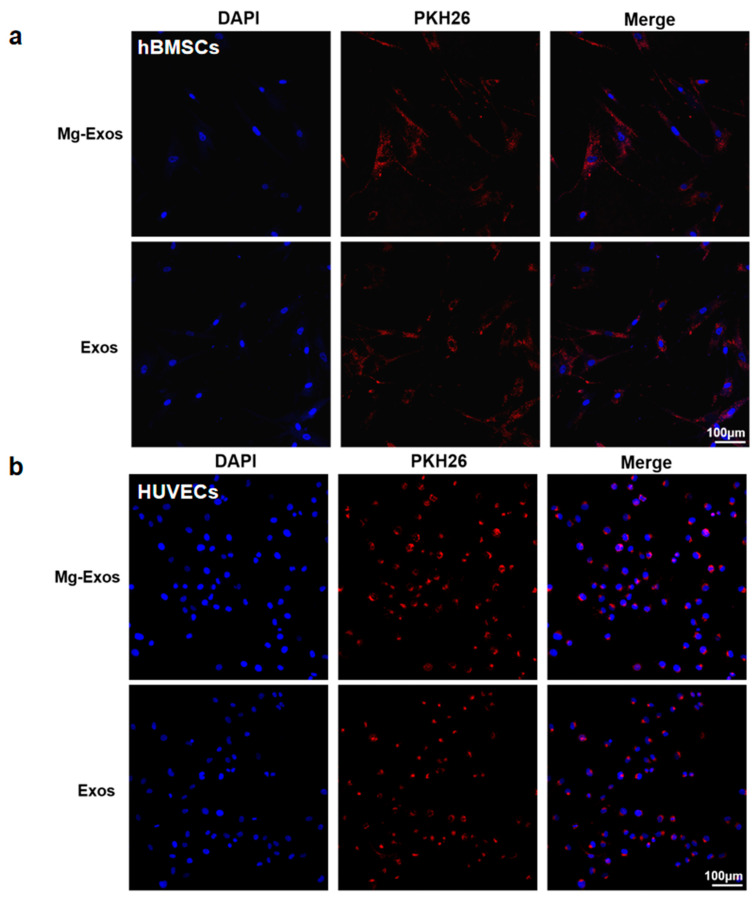
Internalization of Mg-Exos and Exos. Fluorescent images of PKH26-labeled exosomes internalized by hBMSCs (**a**) and HUVECs (**b**). Blue: nuclear. Red: PKH26-labeled exosomes.

Mg^2+^ plays important roles in promoting new bone formation and maintaining vascular function, which also contributes to the restoration of blood supply and bone regeneration in SONFH [41,42,43,44]. However, previous studies have primarily focused on the direct effects of Mg^2+^ or Mg-containing scaffolds on cells such as BMSCs, while the regulatory effects of Mg^2+^ stimulation on the functions of exosomes derived from BMSCs remain unexplored. Studies have indicated that 10 mM Mg^2+^ caused no significant toxicity in BMSCs and exerted the most potent effect in promoting the osteogenic differentiation of BMSCs and upregulating the expression of VEGF [45,46]. In addition, 10 mM Mg^2+^ stimulation optically lowered the anti-angiogenic functionality of macrophage-derived exosomes. Therefore, in this study, rBMSCs were treated with 10 mM MgCl_2_ for 48 h to collect exosomes (designated as Mg-Exos) from the culture supernatant via ultracentrifugation (Figure 2a). Exosomes isolated from untreated BMSCs were designated as Exos. TEM images revealed that the two types of exosomes exhibited cup-shaped or spherical morphologies, with diameters ranging between 50 nm and 150 nm (Figure 2b). NanoFCM indicated that the particle sizes of the two types of exosomes were similar, predominantly distributed within the range of 50–120 nm, with a peak around 60 nm, and the concentrations of the isolated exosomes were approximately ×10^9^ particles/mL for both groups (Figure 2c). Western blot analysis confirmed that both exosomes expressed the specific exosomal markers CD63, TSG101, and HSP70 (Figure 2d). To detect the internalization of exosomes by cells, the two exosomes were labeled with PKH26 and then co-cultured with BMSCs and HUVECs, respectively. Fluorescence microscopy observation showed that the labeled exosomes exhibited homogeneous red punctate fluorescence, which was successfully taken up by BMSCs and HUVECs (Figure 3a,b).

### 3.3. Mg-Exos Promoted Angiogenesis of HUVECs

**Figure 4:**
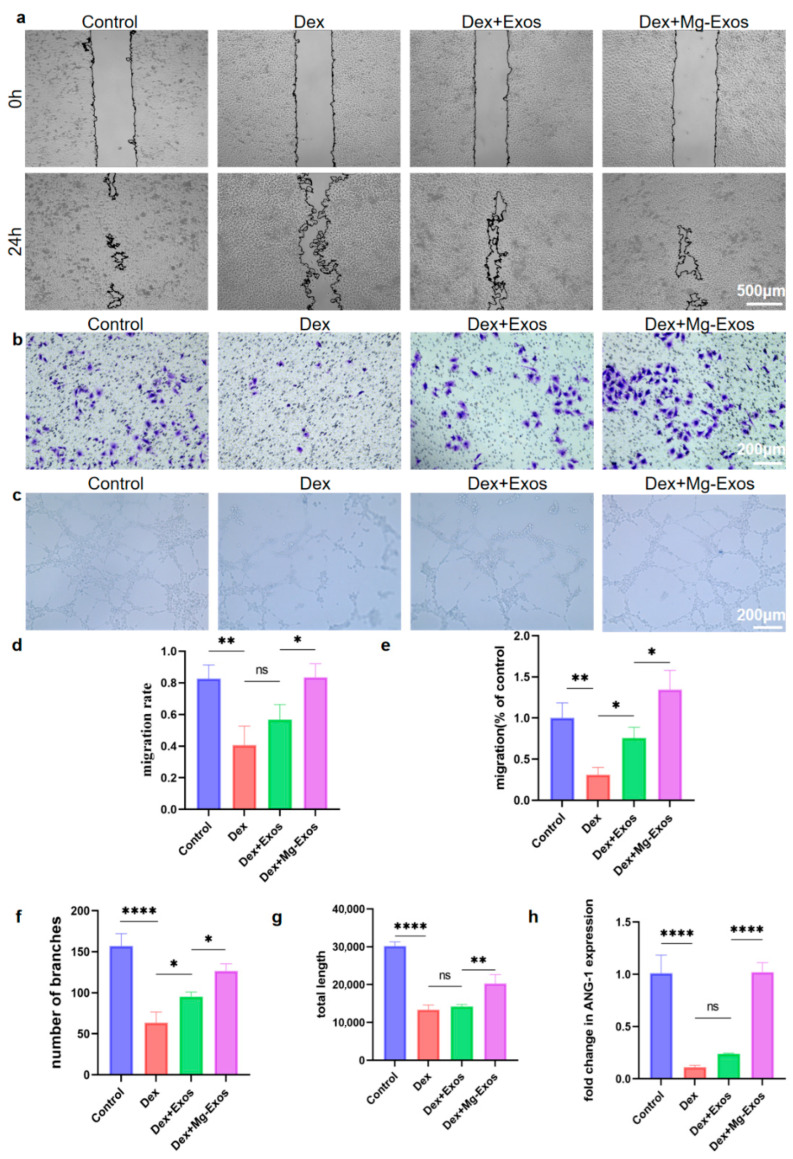
Mg-Exos promoted angiogenesis of HUVECs. (**a**,**b**) Representative images and quantitative analysis of migration of HUVECs determined by wound healing assay (**a**,**d**) and transwell assay (**b**,**e**). Images (**c**) and quantitative analysis (**f**,**g**) of tube formation. mRNA expression level of ANG-1 (**h**). * *p* < 0.05, ** *p* < 0.01, **** *p* < 0.0001, ns: no significant. *n* = 3.

Studies have demonstrated that high-dose glucocorticoid intake impairs endothelial cells, thereby contributing to thrombosis, abnormal thrombin levels, and ultimately, reduced femoral head blood supply, which leads to bone hypoxia, inadequate nutrient supply, and reduced compressive strength, resulting in femoral head collapse [47]. These findings suggest that the recovery of endothelial cell function following glucocorticoid-induced injury could be an effective treatment approach for SONFH. According to previous research, dexamethasone (Dex) at a concentration of 10 μM significantly suppressed the proliferation, migration, and angiogenesis of HUVECs, while also markedly inhibiting the proliferation and osteogenic function of BMSCs [48]. Therefore, we used 10 μM Dex to establish a SONFH cell model to simulate glucocorticoid-induced damage and evaluate the therapeutic efficacy of Mg-Exos. We selected a range of exosome concentrations for initial evaluation using the CCK-8 assay, based on a published study [49]. CCK-8 results showed that cell proliferation was significantly inhibited under Dex treatment. The addition of Mg-Exos and Exos counteracted the damaging effects of Dex on HUVECs, with Mg-Exos exhibiting a stronger protective effect. The most pronounced promoting effect was observed at an exosome concentration of 5 × 10^9^ particles/mL (Figure S1). Hence, this concentration was used in subsequent experiments. The wound healing assay revealed that after 24 h, the migration rate in the Dex group (40.7%) decreased by more than half compared to the Control group (82.7%). Exos partially alleviated the inhibitory effect of Dex on HUVEC migration (56.8%), but the improvement was not significant. In contrast, Mg-Exos almost completely reversed the impact of Dex on HUVEC migration, with a 24 h migration rate reaching 83.6%, similar to that of the Control group (Figure 4a,d). The Transwell assay showed a consistent trend: few cells migrated in the Dex group, while the addition of Mg-Exos reversed the inhibitory effect of Dex (Figure 4b,e). In the tube formation assay, complete tubular structures failed to form after Dex treatment. The addition of Exos improved tube formation, and the Mg-Exos group showed significant increases in total length and number of branches compared to the Dex group, with a better restorative effect than the Exos group (Figure 4c,f,g). qRT-PCR results indicated that Mg-Exos notably enhanced the mRNA expression levels of the angiogenesis-related gene ANG-1 (Figure 4h). In summary, Dex inhibited the proliferation, migration, and tube-forming ability of HUVECs. Exos ameliorated the inhibitory effects of Dex, and exosomes derived from Mg^2+^-stimulated BMSCs exhibited enhanced restorative effects, with a greater promotion of angiogenesis.

### 3.4. Mg-Exos Promoted Osteogenesis of hBMSCs

**Figure 5:**
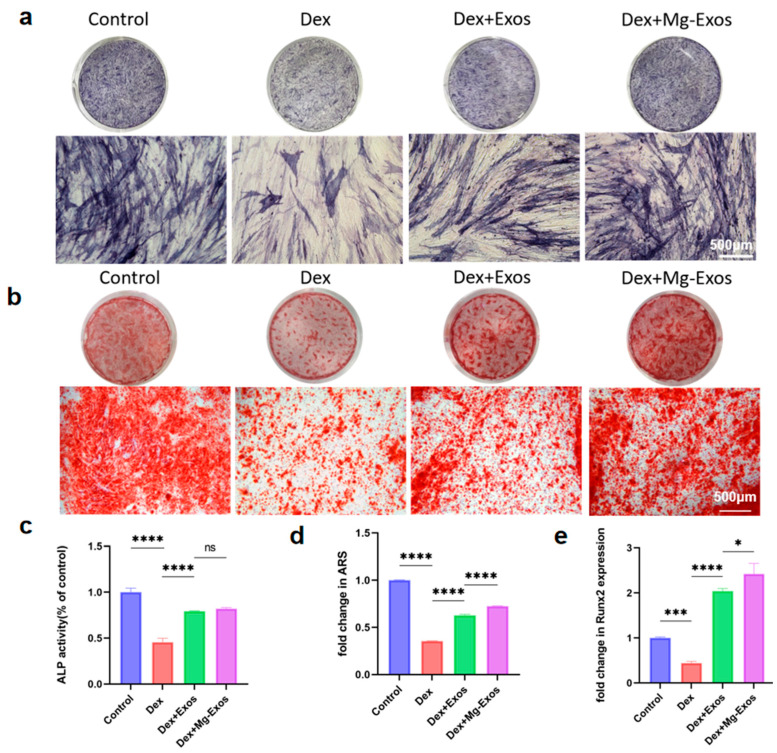
Mg-Exos promoted osteogenesis of BMSCs. Images (**a**) and quantitative analysis (**c**) of ALP staining of BMSCs after 14 days of osteogenic induction. Images (**b**) and quantitative analysis (**d**) of ARS staining of BMSCs after 21 days of osteogenic induction. (**e**) mRNA expression level of Runx2. * *p* < 0.05, *** *p* < 0.001, **** *p* < 0.0001, ns: no significant. *n* = 3.

The decline in osteogenic differentiation capacity of BMSCs induced by glucocorticoids is also a significant etiology of SONFH [50,51]. In this section, we evaluated the influence of Mg-Exos on the proliferation and osteogenic differentiation of Dex-treated hBMSCs. CCK-8 assay results indicated that Dex addition significantly suppressed hBMSCs proliferation, as evidenced by a marked reduction in absorbance at 450 nm after 24 h. Mg-Exos administration partially reversed this inhibitory effect. At a concentration of 5 × 10^9^ particles/mL, Mg-Exos significantly promoted the proliferation of Dex-treated cells. However, increasing the concentration to 5 × 10^10^ particles/mL did not yield a substantially greater enhancement. Therefore, a concentration of 5 × 10^9^ particles/mL was chosen for subsequent osteogenic induction experiments (Figure S2). The osteogenic differentiation capacity of BMSCs was assessed mainly through the expression of the early osteogenic marker ALP and the formation of late-stage calcium deposition. After 14 days of osteogenic induction, ALP staining and quantitative analysis demonstrated that compared to the Control group, the Dex-treated group displayed lighter and more sparse blue-purple BCIP/NBT staining, along with s a significant decrease in ALP expression. In contrast, the Mg-Exos group exhibited intensified staining and higher ALP activity relative to the Dex group. Although ALP expression was elevated compared to the Exos group, the difference lacked statistical significance (Figure 5a,c). This pattern suggests that Mg-Exos may preferentially enhance the late-stage maturation phase of osteogenesis rather than early marker expression. After 21 days of induction, Alizarin Red S staining of calcium nodules and semi-quantitative measurement of OD values after dissolution with 10% CPC demonstrated a consistent trend. The Mg-Exos group exhibited significantly greater mineralized deposition and higher calcium nodule content compared to both the Dex and Exos groups (Figure 5b,d). The pronounced mineralization effect, coupled with the more modest ALP enhancement, collectively indicates a stage-specific enhancement of osteogenic differentiation by Mg-Exos. qRT-PCR analysis performed after 7 days of osteogenic induction with exosome treatment showed that the relative expression of Runx2, a key gene regulating osteogenic differentiation, was reduced by half in the Dex group. Mg-Exos treatment significantly up-regulated the expression level of Runx2 (Figure 5e).

Previous studies have shown the critical role of exosomes in mediating intercellular communication and promoting tissue regeneration [52,53]. Exosomes from mesenchymal stem cells can enhance angiogenesis and osteogenesis under pathological conditions [22,54]. The novelty of our approach lies in the use of Magnesium-primed exosomes, which appear to exert superior protective effects against dexamethasone-induced dysfunction. Magnesium itself has been widely documented for its pro-angiogenic and pro-osteogenic properties [55,56], and our data suggest that these benefits may be partially mediated through exosome-based mechanisms.

Despite these promising results, several aspects of our study warrant further investigation to advance the therapeutic potential of Mg-Exos. Future research could explore whether Magnesium modulates the biogenesis, secretion, or cargo composition of BMSC-derived exosomes, clarifying whether the beneficial effects arise from changes in exosome production or alterations in bioactive molecules such as miRNAs and proteins. In addition, the mechanisms underlying Mg-Exos-mediated osteogenic and angiogenic recovery remain to be fully elucidated; thus, detailed molecular profiling via RNA sequencing or proteomics could help identify key functional components. Furthermore, a thorough biosafety evaluation—including assessments of endotoxin levels, hemocompatibility, and immunogenic potential—will be essential in future preclinical development to complement the current functional findings and ensure translational relevance. Moreover, the absence of in vivo validation represents a significant gap in our study. Subsequent studies should evaluate the efficacy of Mg-Exos in animal models of steroid-induced osteonecrosis. A particularly promising strategy would be to integrate Mg-Exos with biomaterial-based delivery systems—such as hydrogels, scaffolds, or nanoparticles, to achieve sustained release and enhanced localization at the target site [16,57,58].

## 4. Conclusions

In conclusion, our study demonstrates that exosomes derived from Mg-conditioned BMSCs (Mg-Exos) effectively counteract the inhibitory effects of dexamethasone on both the angiogenic function of HUVECs and the osteogenic differentiation of BMSCs. Mg-Exos could enhance osteogenic and angiogenic activities in vitro, and provide a strong foundation for the therapeutic application of Mg-Exos in SONFH. Further mechanistic and in vivo studies will be carried out to validate the results of this study and investigate further the clinical potential of Mg-Exos.

## Data Availability

The original contributions presented in this study are included in the article. Further inquiries can be directed to the corresponding authors.

## Supplementary Materials

The following supporting information can be downloaded at: https://www.mdpi.com/article/10.3390/ma18204687/s1, Figure S1: OD value of HUVECs determined by CCK-8 assay. Figure S2: OD value of BMSCs determined by CCK-8 assay. ** *p* < 0.01, ns: no significant. Table S1: The primer sequences used for qRT-PCR.

## References

[1] van der Goes M.C., Strehl C., Buttgereit F., Bijlsma J.W., Jacobs J.W. (2016). Can Adverse Effects of Glucocorticoid Therapy Be Prevented and Treated?. Expert Opin. Pharmacother..

[2] Motta F., Timilsina S., Gershwin M.E., Selmi C. (2022). Steroid-Induced Osteonecrosis. J. Transl. Autoimmun..

[3] Chang C., Greenspan A., Gershwin M.E. (2020). The Pathogenesis, Diagnosis and Clinical Manifestations of Steroid-Induced Osteonecrosis. J. Autoimmun..

[4] Li Z., Han L., Wang B., Wang P., Wang Y., Wang R., Lv X., Feng Y. (2025). The Role of Piezo1 in Bone Marrow Stem Cells in Response to Elevated Intraosseous Pressure on Regulating Osteogenesis and Angiogenesis of Steroid-Induced Osteonecrosis of the Femoral Head. J. Orthop. Transl..

[5] Cao H., Shi K., Long J., Liu Y., Meng X., Huang C., Hao J., Li L., Zhao Y., Ye T. (2025). PDGF-BB Improves Cortical Bone Quality through Restoring the Osteogenic Microenvironment in the Steroid-Associated Osteonecrosis of Rabbits. J. Orthop. Transl..

[6] Chen Y., Miao Y., Liu K., Xue F., Zhu B., Zhang C., Li G. (2022). Evolutionary Course of the Femoral Head Osteonecrosis: Histopathological—Radiologic Characteristics and Clinical Staging Systems. J. Orthop. Transl..

[7] Cao H., Guan H., Lai Y., Qin L., Wang X. (2016). Review of Various Treatment Options and Potential Therapies for Osteonecrosis of the Femoral Head. J. Orthop. Transl..

[8] Caplan A.I. (2007). Adult Mesenchymal Stem Cells for Tissue Engineering versus Regenerative Medicine. J. Cell. Physiol..

[9] Loebel C., Burdick J.A. (2018). Engineering Stem and Stromal Cell Therapies for Musculoskeletal Tissue Repair. Cell Stem Cell.

[10] Gao X., Ruzbarsky J.J., Layne J.E., Xiao X., Huard J. (2024). Stem Cells and Bone Tissue Engineering. Life.

[11] Wang Y., Wen J., Lu T., Han W., Jiao K., Li H. (2024). Mesenchymal Stem Cell-Derived Extracellular Vesicles in Bone-Related Diseases: Intercellular Communication Messengers and Therapeutic Engineering Protagonists. Int. J. Nanomed..

[12] Li Z., Li Q., Tong K., Zhu J., Wang H., Chen B., Chen L. (2022). BMSC-Derived Exosomes Promote Tendon-Bone Healing after Anterior Cruciate Ligament Reconstruction by Regulating M1/M2 Macrophage Polarization in Rats. Stem Cell Res. Ther..

[13] Dabrowska S., Andrzejewska A., Janowski M., Lukomska B. (2021). Immunomodulatory and Regenerative Effects of Mesenchymal Stem Cells and Extracellular Vesicles: Therapeutic Outlook for Inflammatory and Degenerative Diseases. Front. Immunol..

[14] Ogata K., Katagiri W., Hibi H. (2017). Secretomes from Mesenchymal Stem Cells Participate in the Regulation of Osteoclastogenesis in Vitro. Clin. Oral. Investig..

[15] Lukomska B., Stanaszek L., Zuba-Surma E., Legosz P., Sarzynska S., Drela K. (2019). Challenges and Controversies in Human Mesenchymal Stem Cell Therapy. Stem Cells Int..

[16] Huang J., Xiong J., Yang L., Zhang J., Sun S., Liang Y. (2021). Cell-Free Exosome-Laden Scaffolds for Tissue Repair. Nanoscale.

[17] Bei H.P., Hung P.M., Yeung H.L., Wang S., Zhao X. (2021). Bone-a-Petite: Engineering Exosomes towards Bone, Osteochondral, and Cartilage Repair. Small.

[18] van Niel G., D’Angelo G., Raposo G. (2018). Shedding Light on the Cell Biology of Extracellular Vesicles. Nat. Rev. Mol. Cell Biol..

[19] Kalluri R., LeBleu V.S. (2020). The Biology, Function, and Biomedical Applications of Exosomes. Science.

[20] O’Brien K., Breyne K., Ughetto S., Laurent L.C., Breakefield X.O. (2020). RNA Delivery by Extracellular Vesicles in Mammalian Cells and Its Applications. Nat. Rev. Mol. Cell Biol..

[21] Rani S., Ryan A.E., Griffin M.D., Ritter T. (2015). Mesenchymal Stem Cell-Derived Extracellular Vesicles: Toward Cell-Free Therapeutic Applications. Mol. Ther..

[22] Zhang L., Jiao G., Ren S., Zhang X., Li C., Wu W., Wang H., Liu H., Zhou H., Chen Y. (2020). Exosomes from Bone Marrow Mesenchymal Stem Cells Enhance Fracture Healing through the Promotion of Osteogenesis and Angiogenesis in a Rat Model of Nonunion. Stem Cell Res. Ther..

[23] Zhou Y., Deng G., She H., Bai F., Xiang B., Zhou J., Zhang S. (2023). Polydopamine-Coated Biomimetic Bone Scaffolds Loaded with Exosomes Promote Osteogenic Differentiation of BMSC and Bone Regeneration. Regen. Ther..

[24] Li H., Liu H., Zhou Y., Cheng L., Wang B., Ma J. (2025). The Multifaceted Roles of Extracellular Vesicles in Osteonecrosis of the Femoral Head. J. Orthop. Transl..

[25] Wu J., Wu J., Liu Z., Gong Y., Feng D., Xiang W., Fang S., Chen R., Wu Y., Huang S. (2024). Mesenchymal Stem Cell–Derived Extracellular Vesicles in Joint Diseases: Therapeutic Effects and Underlying Mechanisms. J. Orthop. Transl..

[26] Sadeghi S., Tehrani F.R., Tahmasebi S., Shafiee A., Hashemi S.M. (2023). Exosome Engineering in Cell Therapy and Drug Delivery. Inflammopharmacol.

[27] Wu Z., He D., Li H. (2021). Bioglass Enhances the Production of Exosomes and Improves Their Capability of Promoting Vascularization. Bioact. Mater..

[28] Miceli V., Bulati M., Iannolo G., Zito G., Gallo A., Conaldi P.G. (2021). Therapeutic Properties of Mesenchymal Stromal/Stem Cells: The Need of Cell Priming for Cell-Free Therapies in Regenerative Medicine. Int. J. Mol. Sci..

[29] Lian M., Qiao Z., Qiao S., Zhang X., Lin J., Xu R., Zhu N., Tang T., Huang Z., Jiang W. (2024). Nerve Growth Factor-Preconditioned Mesenchymal Stem Cell-Derived Exosome-Functionalized 3D-Printed Hierarchical Porous Scaffolds with Neuro-Promotive Properties for Enhancing Innervated Bone Regeneration. ACS Nano.

[30] Liu L., Yu F., Chen L., Xia L., Wu C., Fang B. (2023). Lithium-Containing Biomaterials Stimulate Cartilage Repair through Bone Marrow Stromal Cells-Derived Exosomal miR-455-3p and Histone H3 Acetylation. Adv. Healthc. Mater..

[31] Yuan N., Ge Z., Ji W., Li J. (2021). Exosomes Secreted from Hypoxia-Preconditioned Mesenchymal Stem Cells Prevent Steroid-Induced Osteonecrosis of the Femoral Head by Promoting Angiogenesis in Rats. BioMed Res. Int..

[32] Li C., Zhang W., Nie Y., Du X., Huang C., Li L., Long J., Wang X., Tong W., Qin L. (2024). Time-Sequential and Multi-Functional 3D Printed MgO2/PLGA Scaffold Developed as a Novel Biodegradable and Bioactive Bone Substitute for Challenging Postsurgical Osteosarcoma Treatment. Adv. Mater..

[33] Zhang W., Li L., Wang Z., Nie Y., Yang Y., Li C., Zhang Y., Jiang Y., Kou Y., Zhang W. (2025). Injectable and Adhesive MgO_2_-Potentiated Hydrogel with Sequential Tumor Synergistic Therapy and Osteogenesis for Challenging Postsurgical Osteosarcoma Treatment. Biomaterials.

[34] Zhang Y., Li C., Zhang W., Deng J., Nie Y., Du X., Qin L., Lai Y. (2021). 3D-Printed NIR-Responsive Shape Memory Polyurethane/Magnesium Scaffolds with Tight-Contact for Robust Bone Regeneration. Bioact. Mater..

[35] Bai L., Song P., Su J. (2023). Bioactive Elements Manipulate Bone Regeneration. Biomater. Transl..

[36] Zhu Y., Zhao S., Cheng L., Lin Z., Zeng M., Ruan Z., Sun B., Luo Z., Tang Y., Long H. (2022). Mg^2+^-mediated Autophagy-dependent Polarization of Macrophages Mediates the Osteogenesis of Bone Marrow Stromal Stem Cells by Interfering with Macrophage-derived Exosomes Containing miR-381. J. Orthop. Res..

[37] Li X., Zhang Y., Qi G. (2013). Evaluation of Isolation Methods and Culture Conditions for Rat Bone Marrow Mesenchymal Stem Cells. Cytotechnology.

[38] Qin L., Liu N., Bao C., Yang D., Ma G., Yi W., Xiao G., Cao H. (2023). Mesenchymal Stem Cells in Fibrotic Diseases—The Two Sides of the Same Coin. Acta Pharmacol. Sin..

[39] Song K., Huang M., Shi Q., Du T., Cao Y. (2014). Cultivation and Identification of Rat Bone Marrow-derived Mesenchymal Stem Cells. Mol. Med. Rep..

[40] Fang S., Li Y., Chen P. (2018). Osteogenic Effect of Bone Marrow Mesenchymal Stem Cell-Derived Exosomes on Steroid-Induced Osteonecrosis of the Femoral Head. Drug Des. Dev. Ther..

[41] Díaz-Tocados J.M., Herencia C., Martínez-Moreno J.M., Montes de Oca A., Rodríguez-Ortiz M.E., Vergara N., Blanco A., Steppan S., Almadén Y., Rodríguez M. (2017). Magnesium Chloride Promotes Osteogenesis through Notch Signaling Activation and Expansion of Mesenchymal Stem Cells. Sci. Rep..

[42] Bernardini D., Nasulewic A., Mazur A., Maier J.A.M. (2005). Magnesium and Microvascular Endothelial Cells: A Role in Inflammation and Angiogenesis. Front. Biosci..

[43] Gu Y., Zhang J., Zhang X., Liang G., Xu T., Niu W. (2019). Three-Dimensional Printed Mg-Doped β-TCP Bone Tissue Engineering Scaffolds: Effects of Magnesium Ion Concentration on Osteogenesis and Angiogenesis In Vitro. Tissue Eng. Regen. Med..

[44] Lai Y., Li Y., Cao H., Long J., Wang X., Li L., Li C., Jia Q., Teng B., Tang T. (2019). Osteogenic Magnesium Incorporated into PLGA/TCP Porous Scaffold by 3D Printing for Repairing Challenging Bone Defect. Biomaterials.

[45] Qin H., Weng J., Zhou B., Zhang W., Li G., Chen Y., Qi T., Zhu Y., Yu F., Zeng H. (2023). Magnesium Ions Promote In Vitro Rat Bone Marrow Stromal Cell Angiogenesis through Notch Signaling. Biol. Trace Elem. Res..

[46] Yoshizawa S., Brown A., Barchowsky A., Sfeir C. (2014). Magnesium Ion Stimulation of Bone Marrow Stromal Cells Enhances Osteogenic Activity, Simulating the Effect of Magnesium Alloy Degradation. Acta Biomater..

[47] Kang H., Chen H., Huang P., Qi J., Qian N., Deng L., Guo L. (2016). Glucocorticoids Impair Bone Formation of Bone Marrow Stromal Stem Cells by Reciprocally Regulating microRNA-34a-5p. Osteoporos. Int..

[48] Zuo R., Kong L., Wang M., Wang W., Xu J., Chai Y., Guan J., Kang Q. (2019). Exosomes Derived from Human CD34^+^ Stem Cells Transfected with miR-26a Prevent Glucocorticoid-Induced Osteonecrosis of the Femoral Head by Promoting Angiogenesis and Osteogenesis. Stem Cell Res. Ther..

[49] Wu J., Zhang L., Liu H., Zhang J., Tang P. (2023). Exosomes Promote hFOB1.19 Proliferation and Differentiation via LINC00520. J. Orthop. Surg. Res..

[50] Kong L., Zuo R., Wang M., Wang W., Xu J., Chai Y., Guan J., Kang Q. (2020). Silencing MicroRNA-137-3p, Which Targets RUNX2 and CXCL12 Prevents Steroid-Induced Osteonecrosis of the Femoral Head by Facilitating Osteogenesis and Angiogenesis. Int. J. Biol. Sci..

[51] Xu W.-N., Zheng H.-L., Yang R.-Z., Jiang L.-S., Jiang S.-D. (2019). HIF-1α Regulates Glucocorticoid-Induced Osteoporosis Through PDK1/AKT/mTOR Signaling Pathway. Front. Endocrinol..

[52] Deng S., Cao H., Cui X., Fan Y., Wang Q., Zhang X. (2023). Optimization of Exosome-Based Cell-Free Strategies to Enhance Endogenous Cell Functions in Tissue Regeneration. Acta Biomater..

[53] Wang W., Liang X., Zheng K., Ge G., Chen X., Xu Y., Bai J., Pan G., Geng D. (2022). Horizon of Exosome-Mediated Bone Tissue Regeneration: The All-Rounder Role in Biomaterial Engineering. Mater. Today Bio.

[54] Zhu Y., Jia Y., Wang Y., Xu J., Chai Y. (2019). Impaired Bone Regenerative Effect of Exosomes Derived from Bone Marrow Mesenchymal Stem Cells in Type 1 Diabetes. Stem Cells Transl. Med..

[55] Zhu D., You J., Zhao N., Xu H. (2019). Magnesium Regulates Endothelial Barrier Functions through TRPM7, MagT1, and S1P1. Adv. Sci..

[56] Liu W., Guo S., Tang Z., Wei X., Gao P., Wang N., Li X., Guo Z. (2020). Magnesium Promotes Bone Formation and Angiogenesis by Enhancing MC3T3-E1 Secretion of PDGF-BB. Biochem. Biophys. Res. Commun..

[57] Deng L., Liu Y., Wu Q., Lai S., Yang Q., Mu Y., Dong M. (2024). Exosomes to Exosome-Functionalized Scaffolds: A Novel Approach to Stimulate Bone Regeneration. Stem Cell Res. Ther..

[58] Ma W., Yang Z., Lu M., Ma H., Wu C., Lu H. (2024). Hierarchically Structured Biomaterials for Tissue Regeneration. Microstructures.

