# Supplementary material for: Exosomes Derived from Mg-Preconditioned Bone Mesenchymal Stem Cells Promote Angiogenesis and Osteogenesis for Osteonecrosis Treatment
Source: Materials (Basel). 2025 Oct 13;18(20):4687. doi: 10.3390/ma18204687 (PMC12565615; doi:10.3390/ma18204687)
Supplement: Supplementary file 1 [file materials-18-04687-s001.zip › materials-3898223-supplementary.pdf]

Supplementary information

Exosomes Derived from Mg-Preconditioned Bone Mesenchymal Stem Cells Promote Angiogenesis and Osteogenesis for Osteonecrosis Treatment

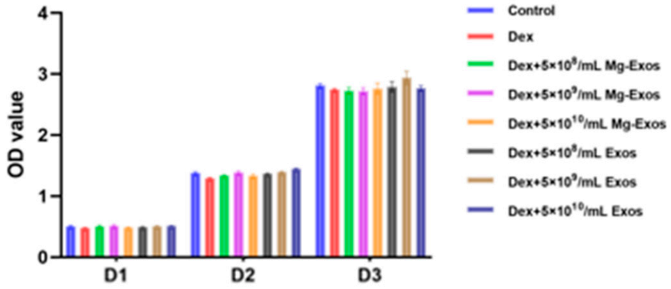

Figure S1. OD value of HUVECs determined by CCK-8 assay.

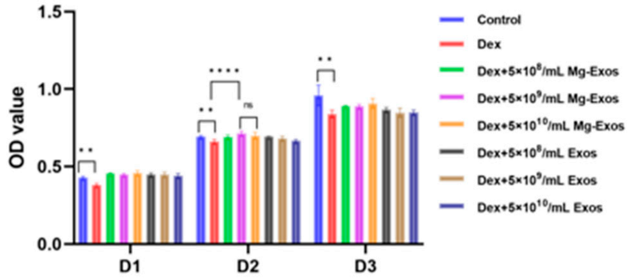

Figure S2. OD value of BMSCs determined by CCK-8 assay.

** $p < 0.01$, ns: no significant

Table S1. The primer sequences used for qRT-PCR.

Gene	Forward primer	Reverse primer
H-ANG-1	5'- AGCGCCGAAGTCCAGAAAAC-3'	5'- TACTCTCACGACAGTTGCCAT-3'
H-Runx2	5'- CTGTCATGGCGGGTAACGAT-3'	5'- GGGTTCCCGAGGTCCATCTA-3'
H-GAPDH	5'- ACAACTTTGGTATCGTGGAAGG-3'	5'- GCCATCACGCCACAGTTTC-3'
